# Reduced renal function is associated with progression to AIDS but not with overall mortality in HIV-infected kenyan adults not initially requiring combination antiretroviral therapy

**DOI:** 10.1186/1758-2652-14-31

**Published:** 2011-06-11

**Authors:** Samir K Gupta, Willis Owino Ong'or, Changyu Shen, Beverly Musick, Mitchell Goldman, Kara Wools-Kaloustian

**Affiliations:** 1 Division of Infectious Diseases, Indiana University School of Medicine, Indianapolis, IN, USA; 2 Moi University School of Medicine, Eldoret, Kenya; 3 Division of Biostatistics, Indiana University School of Medicine. IN, USA

## Abstract

**Background:**

The World Health Organization (WHO) has recently recommended that antiretrovirals be initiated in all individuals with CD4 counts of less than 350 cells/mm^3^. For countries with resources too limited to expand care to all such patients, it would be of value to able to identify and target populations at highest risk of HIV progression. Renal disease has been identified as a risk factor for disease progression or death in some populations.

**Methods:**

Times to meeting combination antiretroviral therapy (cART) initiation criteria (developing either a CD4 count < 200 cells/mm^3 ^or WHO stage 3 or 4 disease) and overall mortality were evaluated in cART-naïve, HIV-infected Kenyan adults with CD4 cell counts ≥200/mm^3 ^and with WHO stage 1 or 2 disease. Cox proportional hazard regression models were used to evaluate the associations between renal function and these endpoints.

**Results:**

We analyzed data of 7383 subjects with a median follow-up time of 59 (interquartile range, 27-97) weeks. In Cox regression analyses adjusted for age, sex, WHO disease stage, CD4 cell count and haemoglobin, estimated creatinine clearance (CrCl) < 60 mL/min was significantly associated with shorter times to meeting cART initiation criteria (HR 1.34; 95% CI, 1.23-1.52) and overall mortality (HR 1.73; 95% CI, 1.19-2.51) compared with CrCl ≥60 mL/min. Estimated glomerular filtration rate (eGFR) < 60 mL/min/1.73 m^2 ^was associated with shorter times to meeting cART initiation criteria (HR 1.39; 95% CI, 1.22-1.58), but not with overall mortality. CrCl and eGFR remained associated with shorter times to cART initiation criteria, but neither was associated with mortality, in weight-adjusted analyses.

**Conclusions:**

In this large natural history study, reduced renal function was strongly associated with faster HIV disease progression in adult Kenyans not initially meeting cART initiation criteria. As such, renal function measurement in resource-limited settings may be an inexpensive method to identify those most in need of cART to prevent progression to AIDS. The initial association between reduced CrCl, but not reduced eGFR, and greater mortality was explained by the low weights in this population.

## Background

Nearly 70% of all HIV-infected individuals globally reside in sub-Saharan Africa, where access to healthcare and, in particular, laboratory services is limited [1]. Despite significant strides in rolling out HIV treatment services to the region, by December 2008, only 44% of individuals requiring HIV treatment based on the 2006 World Health Organization (WHO) criteria (CD4 count under 200 cells/mm^3^, WHO stage 3 disease with a CD4 count under 350 cells/mm^3^, or WHO stage 4 disease) were receiving combination antiretroviral therapy (cART) [2].

In the midst of the region's struggle to provide cART to individuals meeting these conservative criteria for treatment, WHO has recommended raising the CD4 cell count criteria for treatment to 350 cells/mm^3^, as well as treating all individuals with tuberculosis [3]. Many countries are struggling with how to achieve this goal given limited antiretroviral resources, and some are considering targeting specific populations, such as pregnant women and individuals with tuberculosis, as part of the initial phase of this expansion [personal communication: National AIDS Control Program, Republic of Tanzania]. Ideally, countries with resources too limited to expand care to all patients with CD4 counts of less than 350 cells/mm^3 ^would be able to identify and target other at-risk populations.

Renal disease independently predicts progression to AIDS and overall mortality in US urban women not receiving cART [4,5]. In this study of urban American women enrolled in the Women's Interagency HIV Study (WIHS) cohort, Szczech *et al *showed that dipstick proteinuria, but not inverse creatinine, was significantly associated with the development of a new AIDS-defining illness [5]. However, Gardner *et al *[4] found that American women enrolled in the HIV Epidemiology Research Study (HERS) before the availability of cART with either a serum creatinine ≥1.4 mg/dL or proteinuria ≥2+ on urine dipstick had a significantly greater risk of death. Data related to the impact of renal disease on HIV progression and death in African cohorts has been limited to one study from Zambia showing increased 90-day mortality rates after cART initiation in patients with reduced baseline renal function [6]. As such, data related to the ability of renal disease to predict HIV progression and death in untreated HIV-infected African populations is limited.

Although we acknowledge that HIV viral load in combination with CD4 count is likely to be a better predicator of progression than other measures, the availability and cost of viral load testing can be prohibitive in resource-limited settings. Given these constraints, we chose to explore the association between renal disease and HIV disease progression and mortality in sub-Saharan Africans. This study was designed to evaluate this relationship between reduced renal function and HIV disease progression to the 2006 WHO treatment criteria [2], as well as death in a large population of HIV-infected patients not requiring antiretrovirals at enrolment into a care and treatment programme in western Kenya.

## Methods

### Study design

We performed a retrospective analysis of data within the electronic medical records of all patients enrolled into the United States Agency for International Development (USAID)-Academic Model Providing Access to Healthcare (AMPATH) programme from 6 January 2004 (when serum creatinine measurements were routinely performed on all enrollees) until 31 March 2008. Follow up was censored on 18 April 2008. This study was approved by the research regulatory bodies of both the Moi University and the Indiana University Schools of Medicine.

### Study site

AMPATH was initially created as a partnership between Moi University School of Medicine, Moi Teaching and Referral Hospital and a collaboration of North American Medical Schools in November 2001 in order to provide HIV care and treatment in western Kenya [7]. USAID joined the partnership in 2003 when the programme received funding through the US Presidential Emergency Plan for AIDS Relief (PEPFAR). At the end of the study period, the programme was providing HIV care for 52,798 adult patients, of whom 29,124 were on antiretrovirals, at 18 sites throughout western Kenya.

### Study cohort

We included only those individuals who were at least 18 years of age, had not previously received cART, had complete enrolment data available for estimation of renal function (age, sex, serum creatinine, weight) and for other variables of interest (WHO disease stage, haemoglobin, CD4 cell count, eventual initiation of cART), and did not meet USAID-AMPATH requirements for immediate initiation of cART at presentation to care (CD4 count under 200 cells/mm^3^, WHO stage 3 disease or WHO stage 4 disease) [8,9]. We also excluded women who were pregnant at enrolment or who became pregnant during follow up because dates of pregnancy were not uniformly captured in the early years of the AMPATH programme, so we could not confidently attribute pregnancy versus an HIV-related complication as the reason for cART initiation.

### Clinical procedures

At the initial visit, patients undergo a complete history, a complete physical examination, a laboratory assessment (complete blood count, CD4 cell count, Venereal Disease Research Laboratory test (VDRL) and alanine aminotransferase) and a chest x-ray. Serum creatinine is only measured at the enrolment visit. Based on the results of the symptom screen, physical exam and chest x-ray, patients are assigned a WHO stage. Patients not meeting WHO criteria for cART initiation were seen at one- to three-month intervals depending on their co-morbidities. During these visits, an interim history and a symptom-directed exam were performed and CD4 cell counts were obtained every six months. HIV-1 RNA levels were not routinely measured in this cohort due to cost.

An outreach programme is utilized to locate patients who fail to return for their scheduled appointments; however, patients who have been initiated on cART are preferentially traced. As such, there is both active surveillance for death (through the outreach team) and passive surveillance (reports provided to the clinic from family and friends). Data from all visits are recorded on structured patient paper encounter forms and then entered into the AMPATH Electronic Records System by trained data entry clerks [10].

### Statistical analyses

Enrolment renal function was estimated as both creatinine clearance (CrCl) using the Cockcroft-Gault equation [11] and estimated glomerular filtration rate (eGFR) using the 4-variable Modification of Diet in Renal Disease (MDRD) equation [12]. The use of these particular estimating equations and categorizations of CrCl and eGFR were based on recommendations from the National Kidney Foundation [13].

The primary endpoints for these analyses were: (1) time to progression to AIDS, which we defined as meeting WHO requirements for cART initiation (a composite endpoint of developing either a CD4 count under 200 cells/mm^3 ^or developing WHO stage 3 or 4 disease): and (2) time to overall mortality. We specifically chose to use times to meeting criteria for starting cART, rather than actual times to starting cART, as treatment may not have been initiated immediately when criteria were met for a number of logistical and patient-related reasons. Secondary endpoints included time to first CD4 count under 200 cells/mm^3 ^and time to development of WHO stage 3 or 4 disease as separate outcomes as opposed to a composite outcome.

Continuous variables are summarized by medians and interquartile ranges (IQR); categorical variables are summarized by frequencies and percentages. Comparisons of continuous and categorical variables among groups with different renal function parameters were performed with Wilcoxon rank sum test and Chi-square test, respectively. Cox proportional hazard regression models were used to evaluate the associations between renal function and the various endpoints after adjusting for other enrolment covariates that are known to be associated with either HIV disease progression or HIV-related mortality, including WHO stage (1 vs. 2), haemoglobin, CD4 cell count, age and sex. All models were constructed with and without cART initiation as a time-dependent variable.

We chose not to include weight in these initial models as previous studies suggested that the inclusion of weight in the Cockcroft-Gault formula, but not in the simplified MDRD formula, led to significant differences in renal function estimation in HIV-infected sub-Saharan African patients [14]. After we found that there were indeed appreciable differences in renal function estimation between these two formulae and that CrCl, but not eGFR, was significantly associated with overall mortality, we then created weight-adjusted models to determine if weight accounted for these differences in predictive utility. The proportional hazard assumption was tested by the method proposed by Lin *et al *[15]. All analyses were performed using SAS Version 9.2 (Cary, North Carolina). P values less than 0.05 were considered statistically significant.

## Results

### Cohort characteristics

A total of 56,430 adults were enrolled into the USAID-AMPATH programme during the study period. After exclusions due to development of pregnancy during follow up, not meeting study eligibility criteria, and lack of complete enrolment data, 7383 remained for analysis (Figure). This final analysis cohort of 7383 subjects was similar to those excluded for lack of complete data. Specifically, the median (IQR) age and CD4 cell count were 35.5 (29.3-44.0) years and 385 (281-543) cells/mm^3^, respectively, for the analysis cohort, and 36.3 (29.0-42.5) years and 400 (288-561) cells/mm^3^, respectively, for those excluded because of lack of complete data. The percentages of male participants and those with WHO stage 1 disease were 26.9% and 68.0% for the analysis cohort, respectively, and 29.1% and 67.6% for the excluded subjects, respectively.

**Figure 1:**
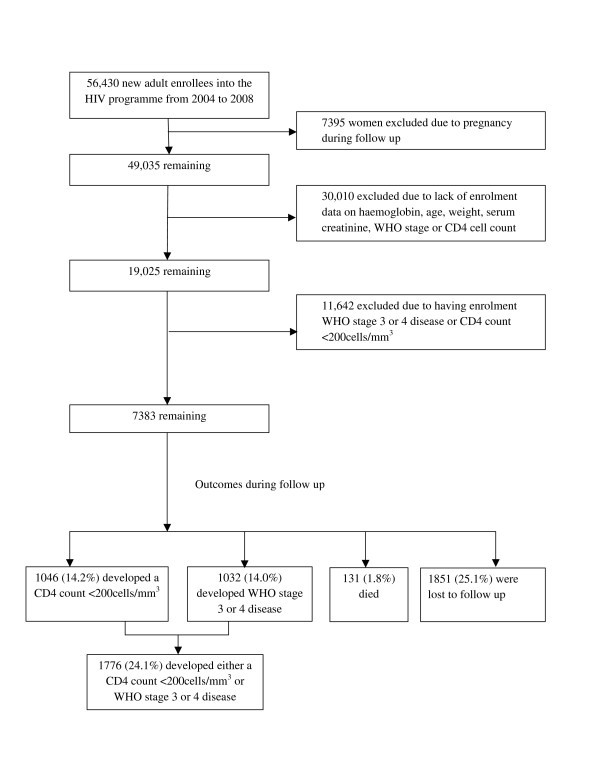
**Selection and outcomes of AMPATH participants in these analyses**.

The median (IQR) duration of follow up for the analysis cohort was 59 (27-97) weeks. As shown in Figure 1, 14.2% of the analysis cohort developed CD4 counts of less than 200 cells/mm^3^, 14.0% developed WHO stage 3 or 4 disease, 24.1% developed either CD4 counts of less than 200 cells/mm^3 ^or WHO stage 3 or 4 disease, and 1.8% died. Of note, the mortality rate in the 4259 subjects who were excluded due to meeting cART initiation criteria at enrolment was 1.4%. A total of 1962 (26.6%) of the analysis cohort initiated cART during follow up. Of these, 47 (2.4%) subjects died after initiation of cART, with the median (IQR) time from cART initiation to death being 19 (7-42) weeks.

Overall, 25.1% were lost to follow up during the study period, which is similar to the lost-to-follow-up rates in other large cohorts in sub-Saharan Africa [16]. Age, haemoglobin, WHO stage, proportions of men, and proportions of those with CD4 cell counts under 350/mm^3 ^were similar between those who were and were not lost to follow up. However, there did appear to be differences in enrolment renal function between these two groups in that 18.5% and 8.3% of those who were not lost to follow up had enrolment CrCl < 60 mL/min and eGFR < 60 mL/min/1.73 m^2^, respectively, whereas 24.9% and 12.4% of those who were lost to follow up had enrolment CrCl < 60 mL/min and eGFR < 60 mL/min/1.73 m^2^, respectively (both p < 0.05).

Table 1 shows the comparisons of enrolment characteristics based on enrolment CrCl or eGFR. The proportions of subjects with renal dysfunction differed based on the estimating equation used. Greater age, having a CD4 count of less than 350 cells/mm^3^, and lower haemoglobin at enrolment were all significantly associated with both a CrCl < 60 mL/min and eGFR < 60 mL/min/1.73 m^2^. Being female was associated with lower eGFR, but not with lower CrCl, at enrolment. Having WHO disease stage 1 (compared with stage 2) at enrolment was associated with lower CrCl, but not with lower eGFR. Lower enrolment weight was associated with lower CrCl, but in contrast, lower weight was associated with higher eGFR. Of note, the median (IQR) number of days between visits for those with and without a CrCl < 60 mL/min in our study cohort were similar at 28 (23-56) and 28 (23-53), respectively. The median (IQR) numbers of days between visits for those with and without an eGFR < 60 mL/min/1.73 m^2 ^were also similar at 28 (23-56) and 28 (25-56), respectively.

**Table 1:** Comparisons of the enrolment characteristics of the analysis cohort by creatinine clearance and estimated glomerular filtration rate categories

		Creatinine clearance (mL/min)^a ^Glomerular filtration rate^b ^(mL/min/1.73 m^2^)^b^					
**Characteristic^c^**	**Total** **(n = 7383)**	**≥60** **(n = 5890; 79.8%)**	**< 60** **(n = 1493; 20.2%)**	**P** **value**	**≥60** **(n = 6689;** **90.6%)**	**< 60** **(n = 694;** **9.4%)**	**P** **value**

Age, years	35.5	34.3	41.8	< 0.001	35.1	39.0	< 0.001
	(29.3-44.0)	(28.7-41.0)	(33.8-49.4)		(29.1-42.6)	(32.1-46.5)	
Female, n (%)	5399 (73.1)	4289 (72.8)	1110 (74.4)	0.24	4851 (72.5)	548 (79.0)	< 0.001
CD4 cell count/mm^3^, n (%)							
>500	2263 (30.7)	1906 (32.4)	357 (23.9)	< 0.001	2075 (31.0)	188 (27.1)	0.005
350-500	1993 (27.0)	1605 (27.2)	388 (26.0)		1821 (27.2)	172 (24.8)	
< 350	3127 (42.4)	2379 (40.4)	748 (50.1)		2793 (41.8)	334 (48.1)	
WHO stage 1, n (%)	5019 (68.0)	4054 (68.8)	965 (64.6)	0.002	4528 (67.7)	491 (70.8)	0.10
Haemoglobin, g/dL	12.6	12.6	12.2	< 0.001	12.6	12.3	0.003
	(10.9-14.0)	(11.0-14.0)	(10.6-13.7)		(11.0-14.0)	(10.6-13.8)	
Weight, kg	59.0	60.0	53.4	< 0.001	59.0	59.8	0.04
	(52.0-65.5)	(54.0-67.0)	(48.0-60.0)		(52.0-65.5)	(53.0-67.0)	
Serum creatinine, mg/dL	0.8	0.77	1.1	< 0.001	0.80	1.4	< 0.001
	(0.7-1.0)	(0.66-0.90)	(1.0-1.3)		(0.68-0.93)	(1.2-1.6)	

### Associations between renal function and cART initiation criteria

Overall, 30.7% and 15.0% of those who eventually met criteria for cART initiation, respectively, had an enrolment CrCl < 60 mL/min and an eGFR < 60 mL/min/1.73 m^2^. As shown in Table 2 (Model 1), our multivariable analyses showed that having an enrolment CrCl < 60 mL/min, compared with an enrolment CrCl ≥60 mL/min, was significantly associated (HR, 1.34; 95% CI, 1.23-1.52; p < 0.0001) with shorter times to meeting cART initiation criteria. Having an eGFR < 60 mL/min/1.73 m^2 ^(Table 3, Model 1) was significantly associated with shorter times to meeting cART initiation criteria (HR, 1.39; 95% CI, 1.22-1.58; p < 0.0001). In both of these models, being male, having WHO stage 2 disease, having a lower CD4 cell count and having a lower haemoglobin level at enrolment were also all independently associated (all p < 0.001) with shorter times to meeting cART initiation criteria. Age was not associated with the primary endpoint in either model. The relationships between lower CrCl or eGFR and times to meeting cART initiation criteria were similar when adjusting for cART initiation.

**Table 2:** Multivariable models showing the hazard ratios for the associations between enrolment creatinine clearance and times to meeting criteria for initiation of cART^a^

	Hazard ratios (95% confidence intervals)	
**Enrolment characteristic**	**Model 1**	**Model 2^b^**

Creatinine clearance^c^(mL/min)		
≥60 (reference)	1.0	1.0
< 60	1.34 (1.23-1.52)^d^	1.24 (1.11-1.39)^d^
Age (per 10 year increase)	1.00 (0.95-1.05)	1.01 (0.96-1.07)
Male sex (compared with female sex)	1.22 (1.09-1.37)^e^	1.27 (1.13-1.42)^d^
WHO stage 2 (compared with stage 1)	1.34 (1.22-1.48)^d^	1.30 (1.18-1.43)^d^
CD4 cell count (per 50 cells/mm^3 ^increase)	0.88 (0.87-0.90)^d^	0.88 (0.87-0.90)^d^
Haemoglobin (per 1 g/dL increase)	0.90 (0.88-0.92)^d^	0.91 (0.89-0.93)^d^
Weight (per 1 kg increase)		0.99 (0.98-0.99)^d^

^a^Combination antiretroviral therapy, defined as development of either CD4 cell count < 200 cells/mm^3 ^or WHO disease stage 3 or 4

^b^Model 1 adjusted for weight

^c^Estimated using the Cockcroft-Gault equation

^d^P < 0.0001

^e^P < 0.001

**Table 3:** Multivariable models showing the hazard ratios for the associations between enrolment estimated glomerular filtration rate and times to meeting criteria for initiation of cART^a^

	Hazard ratios (95% confidence intervals)	
**Enrolment characteristic**	**Model 1**	**Model 2^b^**

Glomerular filtration rate (mL/min/1.73 m^2^)^c^		
≥60 (reference)	1.0	1.0
< 60	1.39 (1.22-1.58)^d^	1.41 (1.23-1.61)^d^
Age (per 10 year increase)	1.03 (0.98-1.08)	1.03 (0.98-1.08)
Male sex (compared with female sex)	1.22 (1.08-1.36)^e^	1.29 (1.14-1.45)^d^
WHO stage 2 (compared with stage 1)	1.35 (1.23-1.49)^d^	1.30 (1.18-1.44)^d^
CD4 cell count (per 50 cells/mm^3 ^increase)	0.88 (0.87-0.90)^d^	0.89 (0.87-0.90)^d^
Haemoglobin (per 1 g/dL increase)	0.90 (0.88-0.92)^d^	0.91 (0.89-0.93)^d^
Weight (per 1 kg increase)		0.98 (0.98-0.99)^d^

^a^Combination antiretroviral therapy, defined as development of either CD4 cell count < 200 cells/mm^3 ^or WHO disease stage 3 or 4

^b^Model 1 adjusted for weight

^c^Estimated using the 4-variable Modification of Diet in Renal Disease Equation

^d^P < 0.0001

^e^P < 0.001

Having a CrCl < 60 mL/min was also significantly associated (p < 0.05) with developing a CD4 count of less than 200 cells/mm^3^. However, in the eGFR model for this outcome, no category of reduced enrolment eGFR was associated with shorter times to developing a CD4 count of less than 200 cells/mm^3^. In the multivariable model examining the associations between enrolment CrCl and the outcome of developing WHO stage 3 or 4 disease, having a CrCl < 60 mL/min (p < 0.001) was associated with shorter times to this outcome. Having an enrolment eGFR < 60 mL/min/1.73 m^2 ^was significantly associated (p < 0.001) with shorter times to developing WHO stage 3 or 4 disease.

We repeated the Model 1 analyses (i.e., without adjustment for weight) with CrCl and eGFR treated as continuous variables (data not shown). Lower continuous CrCl was still significantly associated with shorter times to meeting criteria for cART initiation, time to CD4 cell count of less than 200/mm^3^, and time to WHO stage 3 or 4 disease (all p < 0.03). However, eGFR as a continuous variable was not associated with any of these outcomes.

### Associations between renal function and overall mortality

As shown in Table 4 (Model 1), enrolment CrCl < 60 mL/min was significantly associated with shorter times to overall mortality (HR, 1.73; 95% CI, 1.19-2.51; p < 0.01). In contrast, lower eGFR was not associated with overall mortality (Table 5, Model 1). In both of these models, greater age, being male, having WHO stage 2 disease and lower haemoglobin levels at enrolment were all significantly associated with shorter times to overall mortality (all p < 0.05). Lower enrolment CD4 cell count and initiation of cART were not associated with shorter times to death in either model. These associations were not appreciably altered in models that did not adjust for cART initiation (data not shown). Lower CrCl treated as a continuous variable was not associated (p = 0.07) with time to overall mortality, whereas lower eGFR as a continuous variable was again not associated with overall mortality.

**Table 4:** Multivariable models showing the hazard ratios for the associations between enrolment creatinine clearance and times to overall mortality

	Hazard ratios (95% confidence intervals)	
**Enrolment characteristic**	**Model 1**	**Model 2^a^**

Creatinine clearance^b ^(mL/min)		
≥60 (reference)	1.0	1.0
< 60	1.73 (1.19-2.51)^c^	1.25 (0.84-1.86)
Age (per 10 year increase)	1.22 (1.02-1.45)^d^	1.27 (1.07-1.51)^c^
Male sex (compared with female sex)	1.91 (1.29-2.81)^e^	2.40 (1.61-3.59)^f^
WHO stage 2 (compared with stage 1)	1.54 (1.09-2.18)^c^	1.37 (0.97-1.95)
CD4 cell count (per 50 cells/mm^3 ^increase)	0.96 (0.91-1.01)	0.97 (0.91-1.02)
Haemoglobin (per 1 g/dL increase)	0.76 (0.72-0.81)^f^	0.78 (0.73-0.83)^f^
Initiation of antiretroviral therapy (compared with no initiation)	1.36 (0.91-2.02)	1.35 (0.90-2.01)
Weight (per 1 kg increase)		0.95 (0.93-0.97)^f^

^a^Model 1 adjusted for weight

^b^Estimated using the Cockcroft-Gault equation

^c^P < 0.01

^d^P < 0.05

^e^P < 0.001

^f^P < 0.0001WE

### Influence of weight on the associations between renal function estimates and outcomes

CrCl and eGFR renal function estimates differed in their abilities to predict survival in our study cohort. Because lower weight is itself known to be associated with worse outcomes in HIV-infected patients, we hypothesized that the inclusion of weight in the Cockcroft-Gault equation, but not in the simplified MDRD equation, may explain these differences. To examine this more closely, we then adjusted for weight in our models. Even after this additional adjustment, CrCl was still significantly associated, albeit less so, with shorter times to meeting cART initiation criteria (Table 2, Model 2). In other weight-adjusted models, lower CrCl remained significantly associated with shorter times to developing WHO stage 3 or 4 disease, but was no longer associated with times to developing CD4 counts of less than 200 cells/mm^3 ^(data not shown). Lower eGFR, remained significantly associated with shorter times to meeting cART initiation criteria after adjustment for weight (Table 3, Model 2). In the weight-adjusted survival models, neither lower CrCl (Table 4, Model 2) nor lower eGFR (Table 5, Model 2) were associated with overall mortality.

## Discussion

To our knowledge, the current study is the largest analysis to date investigating the natural progression of HIV disease in sub-Saharan African adults not initially receiving antiretroviral therapy. As such, we could investigate with high confidence multiple predictors of both eventual need for cART and overall mortality.

Our primary goal was to evaluate the utility of renal function to predict HIV-related outcomes. We found that lower renal function, defined either as estimated CrCl < 60 mL/min or as estimated eGFR < 60 mL/min/1.73 m^2^, at enrolment was independently associated with an increased risk of HIV disease progression. Our results differ from the only other study to assess renal abnormalities as predictors for AIDS progression in patients not receiving cART [5].

In analyses of the Women's Interagency HIV Study (WIHS) cohort, Szczech *et al *[5] found that dipstick proteinuria, but not inverse creatinine, was significantly associated with the development of a new AIDS-defining illness. Several reasons may explain the differences in results. The WIHS cohort included only women, whereas our study included both men and women. Differences in diet and environmental conditions may also have contributed to the discrepant results. The definitions of renal function also differed between our analyses. Szczech *et al *used inverse creatinine as a continuous predictor variable, while we used categorical definitions of both estimated creatinine clearances and glomerular filtration rates. Perhaps most importantly, the WIHS cohort analysis could adjust for multiple other potentially confounding variables, including HIV-1 RNA levels, proteinuria, albuminuria and presence of other co-morbidities (hepatitis C co-infection, diabetes, hypertension), which we did not have available in our study cohort.

We did not find in weight-adjusted analyses that renal function was associated with overall mortality. Again, our results conflict somewhat with those from the WIHS analyses, in which inverse creatinine predicted mortality in women who did not receive cART. In addition, Gardner *et al *[4] found that American women enrolled in the HIV Epidemiology Research Study (HERS) before the availability of cART with either a serum creatinine ≥1.4 mg/dL or proteinuria ≥2+ on urine dipstick had a significantly greater risk of death.

The differences between our study and the HERS study may have occurred for similar reasons as noted already between our African cohort and the WIHS cohort. However, in follow-up analyses from the WIHS cohort, Estrella *et al *[17] found that having an eGFR < 60 mL/min/1.73 m^2 ^prior to initiation of cART was associated with higher mortality. In addition, a large Zambian study of nearly 26,000 patients initiating cART [6] found that 90-day mortality rates after cART initiation were significantly higher in patients with reduced baseline renal function. The lack of association between reduced renal function and mortality in those initiating cART in our study may have occurred due to a relative lack of power since only 1946 subjects eventually received cART in our cohort. In our experience, the mortality rates in the proportion of patients who are lost to follow up are significantly higher than those observed among patients retained in care; as such, high rates of loss to follow up may have impacted this outcome [18,19].

The mechanisms by which reduced renal function may lead to faster HIV disease progression are not completely clear. The most likely explanation is that the observed relationships may be confounded by the lack of adjustment for HIV-1 RNA levels and increased systemic inflammation, both of which are related to HIV disease progression and renal function [20-23]. Additional studies that incorporate these HIV disease progression markers are needed to better understand the relationships between renal dysfunction and outcomes in both resource-limited and resource-rich environments.

In patients with low muscle mass, low serum creatinine values may more likely reflect reduced creatinine generation even in the face of renal function impairment. Thus, the use of serum creatinine alone to estimate renal function would not be appropriate for the current study cohort. Given the presence of patients with protein malnutrition and HIV wasting in our cohort (both etiologies of muscle wasting), we chose to use estimated renal function using the two most common equations currently in practice, namely the Cockcroft-Gault equation and the 4-variable MDRD equation, which incorporate variables that should adjust for variability in muscle mass. As such, both equations include not only serum creatinine, but also age and sex. The Cockcroft-Gault equation, in contrast with the 4-variable MDRD equation, also includes weight. Our results demonstrate that the specific inclusion of weight in the Cockcroft-Gault equation greatly influenced the prevalence estimates of reduced renal function estimates in this Kenyan population not yet receiving cART.

Our results corroborate those from another HIV-infected African cohort [14] in which the prevalence of renal dysfunction was much greater when using the Cockcroft-Gault equation compared with the simplified MDRD equation. In addition, adjustment for weight in the CrCl prediction models reduced the association between reduced CrCl and HIV disease progression and completely negated the relationship between lower CrCl and mortality in our study. The importance of weight in our analyses should not be surprising given that lower weight has long been known to be associated with decreased survival in those infected with HIV [24,25]. In addition, it should also be noted that the lack of associations between renal function and outcomes in our models using CrCl and eGFR as continuous variables suggest that the renal function may only be associated with outcomes once a critically low threshold is met and not at higher values.

Several limitations should be acknowledged. As mentioned earlier, the retrospective design relied on using existing data, so several other potential predictors of clinical outcomes, such as HIV-1 viral loads, proteinuria, C-reactive protein, metabolic abnormalities and viral hepatitis co-infection status, could not be studied. Because serum creatinine was not calibrated to the MDRD reference laboratory, bias may have occurred and would limit comparisons with other populations [26]. We acknowledge that missing data, including serum creatinine values, in a substantial number of the USAID-AMPATH enrollees, may limit generalizability. However, the very large sample size of the analysis cohort and its similarity to the excluded patients greatly mitigates this limitation. Also, the results of this study may not extend to those groups who were excluded from these analyses, namely women who became pregnant during the study period. However, we believe our results may be generalizable to other sub-Saharan African cohorts.

In our study, approximately 20% had CrCl < 60 mL/min and 9.4% had stage 3 chronic kidney disease, as defined by the National Kidney Foundation as an estimated eGFR < 60 mL/min/1.73 m^2^. These proportions are similar to published reports of the frequency of renal dysfunction in patients in Zambia, Uganda, and Zimbabwe [6,14]. In addition, our cumulative probability of 22% for meeting cART initiation criteria over the first year is similar to a previous Ugandan study [27] investigating the natural progression of HIV infection to WHO stage 4 disease (26%) for those who had either stage 1 or 2 disease at initial diagnosis. The relatively short follow-up period may have also limited our ability to find significant associations between reduced renal function and mortality in several of our models. Finally, we acknowledge that neither the Cockcroft-Gault equation to estimate CrCl nor the simplified MDRD equation to estimate eGFR has been fully validated in an antiretroviral-naïve HIV-infected population. Thus the accuracy of these estimating equations to reflect true renal function in sub-Saharan African patients is not known.

## Conclusions

In conclusion, we have shown that reduced renal function, estimated as either lower CrCl or lower GFR, in HIV-infected Kenyans not initially meeting cART eligibility criteria was associated with faster HIV disease progression. However, renal dysfunction was not associated with overall mortality in HIV-infected Kenyans. The relatively inexpensive cost for estimating renal function in resource-limited HIV care programmes may be justified in the context of providing additive utility in identifying those who will have faster HIV disease progression and thus require cART more urgently.

Availability of cART is expanding in Kenya, but this availability is not yet sufficient to treat all patients who would otherwise meet current treatment initiation criteria used in resource-rich settings. Thus, identifying even a relatively small proportion of patients (i.e., those with lower renal function) with CD4 counts of more than 200/mm^3 ^and WHO disease stage 1 or 2 would still be beneficial in identifying those who most need cART. Because the simplified MDRD equation to estimate GFR remains independently associated with meeting cART intiation criteria, even when accounting for weight, age, sex and serum creatinine, this equation may be preferable to the Cockcroft-Gault equation as a means to measure renal dysfunction in the context of predicting HIV disease progression. Additional research is needed to understand the mechanisms underlying the associations between renal disease and progression to AIDS.

## Competing interests

The authors declare that they have no competing interests.

## Authors' contributions

SKG conceptualized and designed the study, had primary responsibility for interpretation of the data and drafted the manuscript. WOO assisted in interpretation of the results and provided final approval of the manuscript. CS performed the data analysis, assisted in interpretation of the results and provided final approval of the manuscript. BM assisted with the data analysis, assisted in interpretation of the results and provided final approval of the manuscript. MG assisted in interpretation of the results and provided final approval of the manuscript. KWK assisted with the conceptualization and design of the study, interpretation of the data and drafting of the manuscript. All authors have read and approved the final manuscript.
